# A randomized controlled trial examining Iyengar yoga for young adults with rheumatoid arthritis: a study protocol

**DOI:** 10.1186/1745-6215-12-19

**Published:** 2011-01-21

**Authors:** Subhadra Evans, Laura Cousins, Jennie CI Tsao, Saskia Subramanian, Beth Sternlieb, Lonnie K Zeltzer

**Affiliations:** 1 Pediatric Pain Program, Department of Pediatrics, David Geffen School of Medicine, University of California, Los Angeles, USA

## Abstract

**Background:**

Rheumatoid arthritis is a chronic, disabling disease that can compromise mobility, daily functioning, and health-related quality of life, especially in older adolescents and young adults. In this project, we will compare a standardized Iyengar yoga program for young people with rheumatoid arthritis to a standard care wait-list control condition.

**Methods/Design:**

Seventy rheumatoid arthritis patients aged 16-35 years will be randomized into either the 6-week Iyengar yoga program (12 - 1.5 hour sessions twice weekly) or the 6-week wait-list control condition. A 20% attrition rate is anticipated. The wait-list group will receive the yoga program following completion of the first arm of the study. We will collect data quantitatively, using questionnaires and markers of disease activity, and qualitatively using semi-structured interviews. Assessments include standardized measures of general and arthritis-specific function, pain, mood, and health-related quality of life, as well as qualitative interviews, blood pressure/resting heart rate measurements, a medical exam and the assessment of pro-inflammatory cytokines. Data will be collected three times: before treatment, post-treatment, and two months following the treatment.

**Discussion:**

Results from this study will provide critical data on non-pharmacologic methods for enhancing function in rheumatoid arthritis patients. In particular, results will shed light on the feasibility and potential efficacy of a novel intervention for rheumatoid arthritis symptoms, paving the way for a larger clinical trial.

**Trial Registration:**

ClinicalTrials.gov NCT01096823

## Background

Rheumatoid arthritis (RA) is a chronic autoimmune disorder affecting over 2 million adults in the United States. Symptoms include pain, swelling, stiffness, and loss of joint function. Disability and diminished health-related quality of life (HRQOL) are commonly experienced. While RA is more typically diagnosed in older adults, many young people are also affected. Using an estimated annual prevalence of 1.3% for those under 25 years of age derived from the National Health Interview Survey, close to 400,000 children, adolescents and young adults suffer from arthritis in the U.S. [1].

The economic and personal burden of rheumatoid arthritis is high across adult and pediatric populations [2] but RA may be particularly burdensome to youth. Pain, compromised physical functioning, depression and social isolation commonly occur in affected adolescents [3,4]. Young people with arthritis or rheumatism have increased depression and pain, are more likely to use health-care services, and are less likely to attend school compared to healthy controls and also compared to adolescents with other chronic diseases [3]. An additional concern for young people with RA is the challenge to meet normative developmental milestones, such as independence. Pain and debilitated functioning may leave many in a socially and emotionally compromised state [5]. Such difficulties often persist into later adulthood. Pediatric patients with arthritis are at long-term risk of compromised physical and psychosocial functioning, including decreased rates of employment in the future [4]. Although the maintenance of physical functioning is especially important to prevent future osteoporosis and wheelchair dependency in young RA patients, the realities of mobility limitations often impedes this [6]. Clearly, young people with RA require rehabilitation efforts that target psychological and physical functioning.

Despite the need for physical and psychosocial treatments aimed at adolescents and young adults with RA, traditional approaches remain limited [6]. Current medications, including non-steroidal anti-inflammatory drugs (NSAIDs), disease-modifying rheumatic drugs (DMARDs) and steroid treatments can be useful in lessening pain and stiffness, but adverse events such as gastrointestinal problems commonly occur [7]. Such adverse side effects may compound young patients' already compromised HRQOL. Even for patients who respond well to drugs, pharmacologic therapy is often not enough. Rehabilitation efforts that promote a range of physical outcomes including ambulation, muscle strength and balance are often required to prevent joint weakness, reduced muscle efficiency, and ultimately future disability [6]. Yoga is particularly suited to meeting these rehabilitation needs, as poses designed to increase the strength and mobility of joints and muscles can be individualized to meet each patient's abilities and needs. As reviewed below, yoga has the additional advantage of addressing the patient's psychospiritual functioning, which may further reduce risk of disability.

### Yoga as treatment for chronic conditions

Yoga is a discipline developed in ancient India, characterized as a science of self-study and awareness through *asanas *(body postures), *pranayama *(patterns of breathing), and meditation. Yoga has been embraced in Western settings as a form of exercise and relaxation. Currently, yoga programs are relatively low cost, widely available and safe, when performed properly.

Only a few studies have focused on the impact of yoga on musculoskeletal conditions and none have focused specifically on young people. One study in RA patients did include adolescents but the age range was too broad (aged 15-72 years) to generalize the findings across age groups [8]. Despite the small number of participants in this study (10 in each of the yoga and control groups), yoga significantly improved handgrip strength. Handgrip strength was also significantly improved in 20 patients aged 23-43 years following yoga compared to controls [9]. Garfinkel and colleagues tested IY for 20 patients with osteoarthritis of the hands [10] and 17 patients with carpal tunnel syndrome [11]; both studies resulted in amelioration of pain and improved mobility. In 101 chronic back pain patients (aged 27-57), yoga led to improved back-related functioning, compared to an exercise control group and an education group; at 26 weeks post-intervention the yoga group further demonstrated improved pain bother and functioning compared to controls [12]. In an Iyengar yoga program for 7 patients with osteoarthritis aged 50+ years [13], significant reductions were found across functioning and pain scales. In 60 patients with chronic low back pain aged 23-67 years, Williams et al. [14] found significant improvements for an IYP compared to a control group receiving educational materials; over three quarters reported improved functionality, over half experienced reduced pain, and nearly 90% reduced pain medication.

Kuttner and colleagues [15] provided the only systematic analysis of the use of yoga for pain conditions in children and adolescents (25 IBS patients aged 11-18 years). This intervention consisted of a 4-week home practice of yoga, subsequent to an initial training session. It is unclear to what degree participants actually practiced yoga and to what extent they adhered to the prescribed yoga protocol. Despite these limitations, Kuttner et al. found that the yoga group exhibited significantly reduced IBS symptoms and disability, and improved coping and anxiety relative to wait-list controls.

### Limitations of extant research on yoga for health conditions

Among the most important limitations of the existing work on yoga are: 1) inadequate sample size; 2) overly broad age range (e.g., inclusion of adolescents and elderly patients in the same sample); 3) lack of specification regarding the tradition of yoga utilized for all but five prior studies [10-14]; 4) lack of a theoretical model to inform treatment implementation and assessment of outcomes. Since there is great variation across the numerous traditions of yoga, a lack of standardization can confound interpretations of the overall efficacy of yoga on illness states. Unlike most other studies that failed to specify the school of yogic movement utilized, we will develop an IYP with a specific set of poses that will allow reproducibility. In summary, we will address the above gaps in the literature by testing the efficacy of a standardized IYP (described below) in a well-defined population with appropriate sample size (see Power/Sample Size Calculation). Moreover, we have developed a conceptual model delineating the potential mechanisms related to the IYP to guide our proposed research.

### Biopsychosocial model of disability and pain

**Figure 1:**
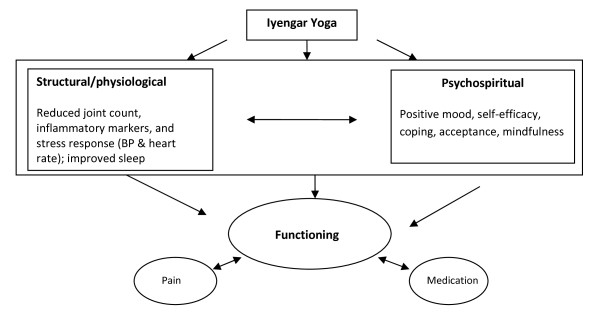
**Conceptual model of Iyengar yoga for RA**.

As a chronic condition, RA is best understood from a biopsychosocial perspective-- i.e., that psychological, biological, and social factors interact in the etiology and maintenance of RA. The biopsychosocial model has been used to understand disability, pain and treatment options for a range of chronic illnesses, including RA [16]. The conceptual model for our current study is similarly built within the biopsychosocial model [17]. It is estimated that psychosocial factors contribute up to 20% of the disability associated with RA [18,19]. Thus, an integrative treatment strategy for patients with RA is warranted. IY is a non-pharmacological rehabilitation intervention that can be combined with a conventional treatment plan. Under the conceptual model (see Figure 1), IY is viewed as impacting physical and psychospiritual well-being. Improvements in these domains in turn can lead to improvements in outcomes. The model offers a guiding heuristic for how we conceptualize the benefits of IYP for RA. We do not propose to test each pathway depicted in this preliminary study nor does the model depict all possible pathways. However, in future larger trials which will incorporate the findings of the present investigation, we plan to refine the conceptual model and test specific pathways by which yoga leads to beneficial outcomes for RA.

### Physiological mechanisms in yoga practice

It is thought that IY quiets the body as well as the mind through attention and relaxation [20]. Hence, yoga has been associated with a number of physical benefits including lowered autonomic arousal (including decreased diastolic and systolic blood pressure and decreased heart rate) [21-23] and a reduction in inflammatory responses in both healthy individuals [24] and those with disease [25]. A recent review concluded that yoga provides psychophysiological benefits that are particularly apparent for people with musculoskeletal conditions [26].

### Psychosocial benefits of yoga

IY has demonstrated positive effects on psychological functioning including increased self-efficacy, or the tendency to persist in health behaviors despite obstacles, and improved coping and mood. In our previous work, we [27] reported reduced depression and anxiety after a 5-week IYP. Similarly, Shapiro [28] found that a 20-week IYP led to reductions in depression, anger, anxiety, and neurotic symptoms in 17 patients (aged 20-71 years). Two other studies found significant improvements in depression, anxiety and self-efficacy following yoga [29,30]. Yoga is also associated with increased coping in patients [15,31-33]. Positive psychology, in turn, is related to better physical functioning [34]. Thus, we expect that yoga will enhance patients' coping, mood and self-efficacy, all of which will impact positively on illness-related functioning. Exercise performed in a group can also promote feelings of social well-being - important for functional status in chronic disease [35]. In future large-scale trials, we will control for such group effects, allowing us to determine the extent to which social benefits of yoga are responsible for improvements.

In contrast to established rehabilitation approaches such as exercise or physical therapy, yoga incorporates spiritual aspects that are purported to confer specific benefits including increased acceptance and mindfulness. IY leads to skill in mastering the challenges of life, which may extend to dealing with chronic illness and pain [26]. It is anticipated that the IYP will enhance patients' compassionate understanding or acceptance of one's health condition, which involves noting the pain without avoidance or limited participation in life goals [36]. Such acceptance appears to be a particularly important facet of quality of life in coping with chronic health conditions [37]. Yoga is also associated with mindful awareness, a concept that has been integrated in empirically validated approaches including cognitive-behavioral therapy [38]. Mindfulness is an openness or receptive awareness to what is occurring in the present. Mindful activity has been found to improve mood and stress [39], likely through strengthening attention on the present [20]. Use and control of attention can be turned towards minimizing stress, disability and pain. It is anticipated that IY will lead to an increase in mindfulness and acceptance which will, in turn, improve functioning among AYA with RA.

### Yoga as an innovative approach

Scientists have long recognized the health benefits of exercise for managing chronic conditions, including RA [40]. In addition to positive effects on structural and physical outcomes, exercise likely impacts brain chemistry to regulate somatomotor-sympathetic circuits and promote psychological health [41]. We propose that yoga shares many of these health benefits, in addition to psychospiritual effects that are specific to yoga practice, including mindfulness and acceptance. A further purported achievement of yoga, but not other forms of physical activity, includes control over physiological systems. Maintaining postures is thought to lead to strengthening and relaxation of voluntary muscles, and eventually to ANS control [42]. Preliminary support for this notion exists with research demonstrating voluntary control over heart rate after a 30-day yoga intervention [43]. Other benefits specific to yoga practice include a non-stressful form of physical activity that promotes an increased alignment of joints and the exploring of conscious and/or unconscious gripping or tension in the body. Such awareness can lead to the release of stress and pain in the body, as well as learning to use bodily systems more effectively. The combination of body, mind and breath awareness in yoga is thought to act on biological and psychospiritual systems to produce homeostasis across multiple aspects of an individual's functioning and physiology [26]. IY thus represents a particularly promising approach for improving RA HRQOL across multiple domains.

#### The specific aims of this research are to

**1**: Determine the feasibility of a six-week twice weekly (12 session) IYP administered in a group format to older adolescents and young adults with RA.

**2**: Compare the impact of IYP on the primary outcomes of functioning and HRQOL to that of a wait-list control group receiving usual care. Functioning will be assessed by two standardized self-report measures, one of general pain-related functioning and the other of arthritis-specific functioning.

**3**: Evaluate the effects of IYP on secondary outcomes of disease activity, pain and mood.

#### The hypotheses to be tested are

**1**: The IYP will be safe, acceptable and feasible: at least 80% of subjects will complete the IYP.

**2**: Following the IYP, participants will show significant improvement on the primary outcomes of general functioning, arthritis functioning and HRQOL relative to controls. The benefits will be apparent post-treatment and at two-month follow-up.

**3**: Following the IYP, participants will report significant improvement on the secondary outcomes of pain, DAS28, and mood compared to controls. These improvements will be evident at both post-treatment and at two-month follow-up.

## Rationale

The proposed study is innovative as it will be the first controlled investigation of a group-based, standardized IYP for older adolescents and young adults with RA. IY is a tradition of yoga that is unique in its emphasis on precise anatomical alignment, use of supportive props, and specific sequences of *asanas *(postures) (see detailed description below). An additional innovation is the use of a mixed-methods approach: patient outcomes and predictors of treatment response will be assessed using both quantitative (standardized questionnaires, physician exam, blood pressure/resting heart rate, assessment of erythrocyte sedimentation rate (ESR) and pro-inflammatory cytokines) and qualitative (interview) methods.

The selection of young patients with RA is based on key considerations. First, young people are likely to be highly motivated as RA impinges upon social, emotional and physical functioning in ways that differentiate them from their peer groups. Second, the stamina/vigor attendant with youth should enhance the ability of young people to practice IY. Third, an age group beginning at older adolescence is generally able to provide their own transportation thereby improving class attendance. Finally, in our previous work we found yoga to be very well-received by young persons [44]. Our preliminary results suggest that yoga is especially well-suited to improving HRQOL and functioning in the proposed age group.

Quality of life and functioning were chosen as the primary outcomes due to the chronic nature of RA and the recent emphasis on patient-reported outcomes in clinical research [45]. HRQOL is perhaps the most significant construct in monitoring a chronic disease's impact [46]. The increased risk of behavioral and emotional disorders faced by youth with chronic illnesses [47] underscores the need to assess and improve HRQOL, including physical, emotional and behavioral functioning. We also intend to examine biomarkers of the disease, including DAS28 scores (see Secondary Outcomes below) and pro-inflammatory cytokine responses before and after the intervention. Cytokines and their receptors likely play a role in the development and maintenance of the inflammatory process in RA [48]. Levels of interleukin-6 (IL-6) and interleukin-1 receptor antagonist (IL-1 RA) are least likely to be affected by biological agents used to treat RA and will be explored as a potential mechanism in the effect of yoga upon patient functioning.

In this study, the control condition will involve patients receiving on-going care as usual for the length of the yoga intervention. While we recognize that the control group will not account for the social aspects of the group-administered IYP, a usual care control group is necessary at this early stage of the clinical trial process. Usual care control groups are appropriate when initially testing novel interventions [49]. If the IY program is found to be superior to the wait-list group in this exploratory study, a future large-scale study will incorporate a control condition that accounts for the social, attention and expectation effects of a group-based intervention.

The overarching aim of the proposed study is to collect preliminary data on the feasibility, safety and efficacy of yoga for RA in patients aged 16-35 years to inform a larger clinical trial. Another key aim of this research is to develop a standardized yoga protocol that may be easily disseminated to other settings for future multi-center studies. The ultimate goal of this research is to begin building a strong evidence base for a readily transportable yoga treatment protocol that may be incorporated within the conventional clinical management of RA.

## Study design and methods

### Overview

This R21 project will consist of three phases: 1) treatment manual development; 2) initial administration and refinement of treatment; 3) a pilot study to evaluate treatment outcome compared to an attention wait-list control condition. These three phases are briefly summarized below.

#### Phase one (3 months)

During this phase, the initial version of the manual will be written. Based on our preliminary studies described above, this manual will describe yoga, breath and meditation exercises that are consistent with specific needs of young RA patients. Attention will also be given to how the poses can best be modified to ensure patients' joints and areas of weakness are protected. The manual will be developed by the study yoga teacher, who will review the intervention with senior Iyengar yoga teachers. During this phase, we will also develop a protocol to evaluate IY instructors' adherence to the manual.

#### Phase two (3 Months)

We will further develop the protocol by conducting an initial IY group. We will also train an additional experienced IY instructor and two assistant instructors. The two IY teachers, with their assistants, will teach a small class of 3 AYA RA patients each (total n = 6) to refine the protocol. The purpose of the assistant instructors is threefold: to promote class safety by assisting patients safely in and out of poses and to record adverse events to bring to the attention of the Safety Board (described below); to take class attendance for determination of patient adherence; and to record the poses and modifications used in each class to be evaluated by the Board (described below) for teacher adherence to the core set of poses. The assistants will note for each student: which poses were performed, which props were used, and any difficulties experienced. Weekly team meetings chaired by the PI will be conducted to solicit suggestions on additional refinements to the protocol.

#### Phase three (18 Months)

**Figure 2:**
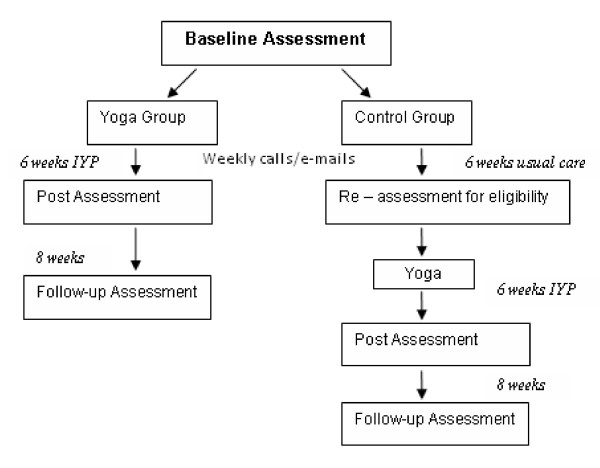
**Flowchart of study design**.

In this final phase, IY teachers will administer the IYP using the revised manual to 70 participants who will be assigned to either treatment (n = 35) or usual care control (n = 35). The controls will be immediately assigned to IYP at the end of the control phase (see Figure 2).

### Design

A randomized control trial (RCT) design will be used to examine the impact of IYP for adolescents and young adults aged 16-35 years with RA on the primary outcomes of general and arthritis-specific functioning, and the secondary outcomes of DAS28, pain and mood.

The overall design of the study is depicted in Figure 2 below. After participants have completed the telephone screening and meet the study's eligibility criteria, they will be randomized into the IYP or usual care control group. All participants will be asked to fill out consent forms before their initial medical assessment, which includes a phlebotomy, measurement of blood pressure/resting heart rate, and a joint count conducted by a physician who will be kept unaware of group assignment. Before the medical assessment, a research assistant will send a link to the questionnaires for participants who choose to complete them online. A hard copy of the questionnaires will be taken to the medical assessment if the participant chooses to complete the paper-based version in a private medical office.

Those assigned to the IYP will then engage in the 6-week program; they will repeat the baseline assessments immediately following treatment and again at a two-month follow-up. Patients initially assigned to the wait-list group will continue their usual care. Both the yoga and control groups will either receive weekly phone calls or e-mails to monitor symptoms, medication use and physical activity. After 6 weeks, control participants will be re-assessed to ensure that they still meet study eligibility criteria; those that continue to meet criteria will then take part in the IYP. Following the IYP, participants will then repeat the baseline assessments at post-treatment and two-month follow-up.

### Enrollment criteria

Young women and men will be eligible for the study if they meet the following criteria:

• Age 16-35 years.

• Diagnosis of RA, according to the revised 1987 ACR criteria for at least 6 months.

• Concomitant use of disease modifying antirheumatic medications (hydroxychloroquine, sulfasalazine, methotrexate, leflunomide, etc.) and/or biologic agents (infliximab, etanercept, adalimumab, abatacept, rituximab, anakinra) is permitted provided that the dose(s) have been stable for 8 weeks prior to screening, and subjects may reasonably be expected to remain on stable doses throughout the study.

• Concomitant use of NSAIDs and low dose corticosterioids (e.g., prednisone at doses of 10 mg/day prednisone or equivalent) is permitted provided that the dose(s) have been stable for 4 weeks prior to screening, and subjects may be expected to remain on stable doses throughout study duration.

• Disease activity, as defined using a 28 joint count by ≥ 5 tender joints, ≥ 5 swollen joints, and one of the following: Erythrocyte Sedimentation Rate (ESR) ≥ 28 mm/hour, C-reactive protein (CRP) ≥ 1.5 mg/dL, duration of a.m. stiffness ≥ 45 minutes.

• Able and willing to give written informed consent and comply with the requirements of the study.

• Ability to speak and understand English.

### Exclusion criteria

Patients will be excluded from the study based on the following criteria:

• Intra-articular steroid injections within 4 weeks of screening.

• Treatment with any investigational agent within 8 weeks of screening or 5 half-lives of the investigational drug (whichever is longer).

• History of drug, alcohol, or chemical abuse within 6 months prior to screening.

• Any other disease, metabolic dysfunction, physical examination finding, or clinical laboratory finding giving reasonable suspicion that might affect the interpretation of the results or render the patient at high risk from treatment complications.

• Inability to comply with study and follow-up procedures.

• Currently pregnant.

• Previous practice of Iyengar yoga within the past three months.

• Inability to speak and understand English.

According to the Power/Sample Size calculations outlined below, we will recruit 35 patients each for the IYP and control conditions. After accounting for our projected attrition rate of 20%, we estimate there will be roughly 28 treatment completers in each condition. To accommodate 56 participants during the 36-month study period, we plan to conduct 4 initial IYP groups and 4 initial control groups, with approximately 8 patients in each group.

### Sample Size Determination and Power Analysis

A power analysis was conducted to determine the appropriate sample size. Using a pre-test post-test for randomized groups, calculating power of the interaction of treatment by time, the sample size estimate is a total sample of 54, or 27 subjects per group (at a 0.05 significance level, 80% power for a medium effect size). We calculated a medium effect size given that our recent single-arm pilot study found an effect size of .46 (Cohen's d) for the HAQ, our arthritis-specific primary outcome measure. Assuming a 20% attrition rate that is consistent with our previous yoga studies, we will recruit 35 participants for each group (total n = 70).

### Randomization

Before completing the baseline assessments, participants will be randomly allocated to the intervention or control group. The research team will assign the patient to the groups using randomization schedules generated by UCLA Biomathematics for group assignment in blocks of 4. As each patient is enrolled, he or she will be assigned to the next number on the list and the associated condition, yoga or usual care.

### Recruitment

Participants will be recruited from the UCLA Division of Rheumatology, the UCLA pediatric rheumatology program, private medical offices of rheumatologists, UCLA Student Psychological Services, health newsletter ads, the young adult support network of the Southern California Chapter of the Arthritis Foundation, and community resources, such as library and other public notice boards, as well as websites, such as Craigslist. The UCLA Division of Rheumatology has a current patient population of 150 individuals with RA who are 16-21 years of age. The UCLA pediatric rheumatology program sees 100 patients with arthritis 16 years and older. Private practice rheumatologists in Los Angeles who have agreed to assist with our project can refer an additional 200 patients with RA in our targeted age range. The Southern California chapter of the Arthritis Foundation will help us recruit from their pool of several hundred RA patients who regularly receive their email announcements and mailed bulletins. In all, we anticipate being able to recruit from a pool of over 500 individuals with RA in the targeted age-range.

The project director will meet with health care providers, staff and support group leaders to inform them about the project and to ask them to distribute and post flyers about the study. The flyers will describe the project and ask interested volunteers to call the project manager to establish their eligibility. Additionally, a lay advisory board comprised of young adults with RA will be formed and consulted with in the development of recruitment materials as well as in the finalization of the protocol and research instruments.

Effort will be made to recruit the patient sample to be broadly representative of the general population of RA patients in the targeted age range. Epidemiological data suggest that the racial composition of RA in the U.S. is approximately 84.4% non-Hispanic whites, 8.3% non-Hispanic blacks, 2.3% Mexican American and 5% other [50]. Women are between 60-75% more likely to experience RA than men [50,51]. We will also attempt to recruit patients to reflect the demographic composition of the Greater Los Angeles area which is estimated to be approximately 75% White, 13% Asian, 10% African-American, 1% American Indian/Alaska Native, and 1% Native Hawaiian/Other Pacific Islander; ethnic makeup will be 45% Hispanic and 55% Non-Hispanic.

### Intervention

#### Iyengar Yoga Program (IYP)

**Figure 3:**
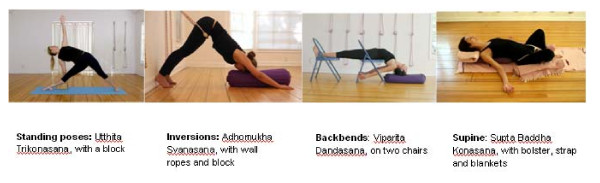
**Selected yoga poses**.

IY is a traditional form of yoga taught in the lineage of BKS Iyengar, who is known for his prominent texts on yoga and innovative teaching techniques. Iyengar has developed specific methods of teaching therapeutic yoga practices to people with health problems [52-54]. IY is an ideal form of activity for people with RA, as the emphasis on alignment in this practice protects joints, is unlikely to irritate inflamed joints and involves sufficient movement to tap the beneficial effects of exercise. Young RA patients with fatigue, restricted motion and painful joints can perform postures with the support of props (blocks, bolsters, chairs, straps and blankets) that allow postures to be held without stress. Yoga postures are designed to promote circulation, stimulate the hormonal system and circulate lymph through body position and muscle activity. The use of props allows inverted postures to be held for long periods of time without fatigue so that the lymph is well circulated without strain. Challenging back bending postures designed to stimulate the adrenal glands can also be held for longer periods of time with the support of props. The poses to be included in this intervention are based on the teachings of BKS Iyengar. The poses include supine poses, passive backbends, standing poses, seated poses, forward bends and supported inversions. All will be done with use of props as needed (see Figure 3).

Yoga taught in the Iyengar tradition is known for an extensive teacher training program. Iyengar teachers study for at least seven years before being certified to work with students who have therapeutic needs. They must pass several certification tests before an impartial national board. These tests include teaching, performance of poses and breathing techniques, anatomy and therapeutic sequences. Because of this training, IY teachers across the country and around the world can accurately reproduce the IYP in this study.

The proposed IYP will be administered twice a week for 1.5 hours duration per session for 6 weeks. Due to the extensive time commitment required for the IYP, there will be no instructions for home practice, but neither will participants be discouraged from practicing at home. At the end of the 6-week IYP, those participants who ask for referrals to IY studios/teachers in the community will be given a list of referrals. All home practice as well as post-IYP yoga classes will be monitored during the weekly phone calls/e-mails and at the post-treatment and follow-up assessments; the extent of home practice will be evaluated in the analyses for its potential impact on outcome.

Should subjects experience a flare-up during the IYP, the research team will consult with the patient's rheumatologist and a modified version of the IYP will be offered to the subject consisting of restorative poses that do not place strain on the affected joints.

### Treatment administration

**Figure 4:**
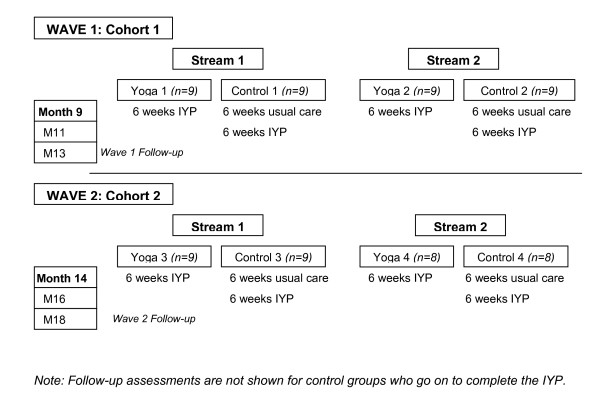
**Group entry timeline**.

As illustrated in Figure 4, two streams of yoga classes will run concurrently to minimize the overall length of the study and to maximize the likelihood that yoga classes will be convenient for patients' schedules. For example, Stream 1 classes will be held on Mondays and Wednesdays, while Stream 2 classes will be held on Tuesdays and Thursdays. Stream 1 and Stream 2 will comprise the first Wave of patients (Wave 1). Upon the completion of Wave 1, a second wave of patients (Wave 2) will consist of two additional Streams of classes. A make-up class will be scheduled each week for any patient not able to attend their usual session. Thus, all patients should be able to receive the full course of IYP since classes will be run 5 days per week (e.g., make-up classes held on Fridays). We will make note of how many make-up classes each patient attends and test for possible differential effects due to missing regular classes. Patients in the control groups will be given the opportunity to take part in the IYP at the end of the 6-week control period. To increase patient safety and accurate practice of poses, IY classes will hold a maximum of 9 patients.

### Usual care control

Patients initially assigned to this condition will be instructed to continue with their on-going medical care. During the control period, these patients will be telephoned or emailed by study personnel to complete the weekly monitoring form (described below) to monitor symptoms, functioning and mood.

## Primary outcomes

**1. *Health Related Quality of Life - Short Form-36 ***(SF-36) [55] is a generic core HRQOL measure yielding an 8-scale profile of functional health and well-being. The SF-36 performs comparatively better than other HRQOL measures in terms of reliability, validity and lightness of respondent/administrative burden [56]. It can be completed in 5-10 minutes and has been used with children as young as 10 years of age [57].

**2. *Pain-related functioning ***will be measured by the ***Pain Disability Index ***(PDI) [58]. The PDI assesses the impact of pain on ability to participate in basic life activities, including social activity, sexual behavior, self-care and life-support activity. The PDI has been used with patients as young as 15 [59]. Good internal reliability (α = .82) and validity have been reported [58,60]. It takes less than 5 minutes to complete.

**3. *Arthritis functioning ***will be assessed with the ***Health Assessment Questionnaire ***(HAQ) [45]. Items include questions about dressing and grooming, rising, eating, walking, hygiene, reaching, grip and activities. The HAQ is one of the most widely recognized measures of patient functioning, with acceptable reliability and validity [45]. It has been used successfully with adolescents as young as 13 years of age [61].

## Secondary outcomes

**1**. ***Disease Activity Scale (DAS) 28 ***is a combined index that measures disease activity in patients with RA and remains a more thorough, established alternative to standard medical exams. This index includes a 28 tender joint count, 28 swollen joint count, Erythrocyte Sedimentation Rate (ESR) and general health assessment using a visual analogue scale. The ESR indirectly measures inflammation in the body and involves collecting blood samples, which will be performed by a qualified phlebotomist.

**2**. ***Pain ***will be measured with a ***numeric rating scale ***(NRS) that asks on a scale of 0 (no pain) to 10 (worst pain possible), what is your current level of pain?

**3**. ***Mood ***will be evaluated using the ***Brief Symptom Inventory 18 ***(BSI-18) [62]. Respondents rate how often they have experienced anxiety, somatization and depressive symptoms within the past 7 days on a 5-point Likert scale ranging from 'Not at all' to 'Extremely.' The BSI-18 has shown adequate to good internal consistency (α range = .74 - .89) and validity [62]. The BSI-18 requires a 6^th ^grade reading ability and takes approximately 4 minutes to complete.

### Exploratory variables

**1. *Blood Pressure and Resting Heart Rate ***will be taken at each medical assessment to evaluate changes in autonomic nervous system activity using a non-invasive, portable, digital blood pressure monitor. A mean blood pressure/resting heart rate value will be derived by taking 2 readings, each 1 minute apart.

**2. *Cytokine Assays ***will be collected from subjects before and after the intervention, at the same time that the blood draws for ESR will be carried out. For the evaluation of pro-inflammatory cytokines, interleukin1-receptor antagonist (IL-1 RA), interleukin-6 (IL-6) and isolated PBMC will be incubated with LPS (500 μl) to yield a final concentration of 5 μg/ml (Ackerman et al., 1998). This dose of LPS has been found to yield optimal production of IL-1, and IL-6 following a 48-hour incubation. Concentrations of these pro-inflammatory cytokines from stimulated cell cultures will be determined by ELISA (R & D Systems). For IL-6, the ELISA intra-assay CV% is 3.1, inter-assay CV% is 2.5, sensitivity <0.7 pg/ml; for IL-1, the intra-assay CV% is 2.3, inter-assay CV% 4.1, sensitivity <1.0 pg/ml. For these assays, we will use high sensitivity kits. The subjects will be brought to a hospital laboratory where a trained phlebotomist will conduct the blood draws needed to assess disease activity in participants. For participants in the intervention group, these blood draws will be performed during a baseline assessment and again at the end of the yoga intervention. Wait-list controls will have their blood drawn at study entry, as well as before and after the yoga intervention.

**3. *Arthritis Self-efficacy Scale ***(ASES) [63] is designed to assess arthritis patients' beliefs that they can perform specific tasks or behaviors to cope with the consequences of chronic arthritis. The scale has 20 items to measure three subscales: pain, function and other symptoms. Reliability is good (α range = .76 - .89). The scale takes approximately 5 minutes to complete.

**4. *Coping Strategies Questionnaire ***(CSQ) [64] is a 42-item checklist measured on a 0-6 Likert scale designed to measure coping in relation to pain. Validity and reliability has been supported in a number of different pain populations, including arthritis patients [64-67]. A child version of the scale (CSQ-C) [34] has been validated and will be used for patients aged 16-20 years. The questionnaire takes approximately 10-15 minutes to complete.

**5**. The ***Chronic Pain Acceptance Questionnaire ***(CPAQ) [68] is a 20-item measure with two subscales: Activity Engagement assessing patients' participation in activities regardless of pain and Pain Willingness, assessing absence of attempts to control or avoid pain. The CPAQ has acceptable reliability (*α*=.78-.82) and validity [68]. It takes approximately 5-10 minutes to complete and is suitable for older adolescents as young as 16 years of age.

**6. *Pittsburgh Sleep Quality Index ***(PSQI) [69] assesses subjective sleep quality over the past month. This 19-item index measures seven components: subjective sleep quality, sleep latency, sleep duration, habitual sleep efficiency, sleep disturbance, use of sleeping medications and daytime dysfunction. Individual component scores or global scores ranging from 0-21 can be evaluated with higher scores indicating poorer sleep quality. The overall reliability coefficient (Cronbach α) is 0.83 and test-retest reliability has a Pearson correlation of 0.85. The index can be completed in less than 5 minutes.

**7. *Functional Assessment of Chronic Illness Therapy Fatigue Subscale ***(FACIT-Fatigue) [70] includes 13 items that assess physical and functional consequences of fatigue. Scores range from 0-52 on a reverse 4-point Likert scale, with higher scores indicating less fatigue. The FACIT-Fatigue has excellent test-retest reliability (*r *= 0.90) and internal consistency (α = 0.93-0.95).

**8. *Mindfulness ***will be assessed using the Five Factor Mindfulness Questionnaire (FFMQ) [71,72], a 39-item self-report measure derived from a factor analysis of items from previously developed mindfulness questionnaires. Items are rated on a Likert scale ranging from 1 ('never or very rarely true') to 5 ('very often or always true'). There are five subscales relating to the five facets of mindfulness: Observe, Describe, Act with Awareness, Nonjudging and Nonreactivity. The FFMQ has been shown to have good internal consistency, with alpha coefficients ranging from .75 to .91, and relationships between the facet scales and other variables are consistent with predictions in most cases. It has been used with high school-aged students previously [73]. It takes less than 10 minutes to complete.

**9. *Medication use ***will be assessed during the patients' weekly phone call/e-mail during which the ***Weekly Monitoring Form ***will be administered. These weekly forms will be used to gather ratings of patients' pain, fatigue, anxiety and depression using 0-10 scales; we will also assess the extent of physical activity during the past week and the extent (i.e., frequency/duration) of any home practice of yoga.

A ***weekly monitoring form ***will include specific questions about functioning, mood, pain, improvement, activity and medication use. The form is an important tool as patients' self-reported functioning will form the basis for understanding the action of yoga over time on symptoms. Thus, responses to the form will be used to determine the minimum and maximum number of yoga sessions needed to produce change. For participants who prefer completing this form online, an online link will be sent to them each week. Otherwise, the form will be telephone-administered by a trained research assistant once a week for two weeks preceding the intervention to ascertain baseline functioning, and then once a week during the intervention and control periods.

## Qualitative measures

While the quantitative data we propose to gather and analyze should provide us with valuable information as to the extent to which the IYP is impacting quality of life and ability to function, qualitative interview data should also serve to elucidate the quantitative data and help us to tease out the nuances of the findings. That is, we expect the semi-structured interviews to yield a rich source of information that provides us with a more comprehensive picture of the issues facing RA patients and the process by which the yoga practice changes their daily lives. Specific topics to be covered will include: the nature of their symptoms and the life domains they affect; how specifically and the extent to which these life domains are impacted; the patients' attempts at self-management of the symptoms; the strength of their social networks and the impact of the symptoms on these networks; and their perceptions of the yoga program. Interviews will be conducted at the end of the intervention by a social scientist experienced in the design, collection, and analysis of qualitative data.

## Data analyses

### Quantitative statistical analysis

After the data have been entered and cleaned, the statistical plan will unfold in two stages. The first stage will involve assessing outcome measures for reliability and general integrity (e.g., distribution characteristics) to ensure that appropriate techniques are applied during data reduction and model testing. The second stage will consist of: 1) descriptive procedures to detail the dataset, and 2) primary analyses to answer the research questions forming the basis of this proposal.

Each stage of data analysis detailed below will initially be carried out separately for younger (16-18 yrs) and older (19-21 yrs) sub-groups. If no differences are found on analyses, the groups will be combined.

### Preliminary analyses

Once the data set has been cleaned and examined for skewness and missing data, exploratory data analyses will be conducted using univariate methods to describe the variables of interest and bivariate techniques to characterize their interrelationships within our sample. Continuous variables will be summarized over time in each group using means and medians and we will also report their range, interquartile range and standard deviations. Cross tabulated frequencies will be given for all discrete variables by group and time. Results from these descriptive analyses will be used to guide the primary analyses by providing information about potential problem measures (e.g., heavily skewed distributions) or problematic variable relationships (e.g., highly correlated predictors) and will help determine if transformations of some outcomes are possible in order to use parametric methods. We will test for baseline between-group differences in the outcome measures. Any variables found to vary significantly by group will be included as covariates in primary and secondary analyses. This will also serve to verify that the randomization was successful.

Missing data will be handled using multiple imputation [74] using the SAS routines PROC MI (20 imputations) and PROC MIANALYZE (to combine estimates). The imputation process is fostered by including additional variables (not part of the intended analytic model) that are highly correlated with variables that have missing data or that are associated with the probability that those variables have missing data. Such covariates (used in imputation only) will vary from analysis to analysis and their viability will be evaluated by preliminary analysis (e.g., zero-order correlations with variables with missing data) and their impact on the imputation model (e.g., change in standard errors of imputed variables). Multiple imputation has been shown to perform well with small samples [74].

For ***Hypothesis 1***: The IYP will be safe, acceptable and feasible: more than 80% of subjects will complete the training program, we will examine the frequencies of drop-outs for the IYP and control groups. We will compare attrition rates between the groups; we will also compare demographic characteristics and baseline values of functioning between those who complete the protocol and those who do not. If drop-outs are random, then the results may be considered unbiased. If drop-outs are not random, we will comment on the direction and magnitude of potential biases.

### Primary analyses

To test ***Hypothesis 2***: Participants in the IYP will demonstrate significantly greater improvement in HRQOL and functioning compared to controls. The primary outcomes include the SF-36, PDI and the HAQ. Two sets of analyses will be conducted. The first set of analyses will compare the initial yoga and control groups immediately after the IYP and control phase, respectively. Separate mixed group (yoga vs. control) by time (baseline vs. post-treatment/post-control) repeated measure analysis of variance (ANOVA) will be conducted on the primary outcomes, or the non-parametric equivalent (Friedman procedure), in the event the assumptions for parametric analyses are not met. We will use the Tukey-Fisher criteria for post hoc mean/median comparisons under the ANOVA or Friedman model. Consistent with ***Hypothesis 2***, we expect a group by time interaction wherein the yoga group will report improved functioning from baseline to post-treatment compared to the control group. The second set of analyses will assess maintenance of treatment gains during the follow-up period. If yoga and control groups do not differ significantly on SF-36, PDI and HAQ scores at baseline, then data from the two groups will be combined in order to maximize power. If groups differ at baseline, each group will be analyzed separately. Changes in SF-36, PDI and HAQ scores across baseline, post-treatment and follow-up will be evaluated using separate within-group repeated measures ANOVAs; Friedman's procedure will be used if parametric assumptions are not met. Consistent with ***Hypothesis 2***, we expect significant improvements in functioning scores from baseline to post-treatment, persisting at follow-up.

We will also conduct an ***intent-to-treat (ITT) analysis***on primary outcomes to confirm our findings in completers by including all participants who enrolled in the study, using the last observation carry forward approach. Participants who drop out will be included in the analysis by utilizing outcome scores before attrition.

### Secondary analyses

To test ***Hypothesis 3***: Participants in the IYP will report significantly less pain, improved joint count, and mood compared to controls. The same methods used for the primary analyses will be used for the secondary analyses on pain intensity (averaged across the weekly NRS ratings taken during the initial 6-week IYP/control period) and mood. Consistent with ***Hypothesis 3***, we expect that the yoga group will evidence greater improvements on these measures over the 6-week treatment period relative to controls. We will also conduct similar within-group repeated measures analysis, as described in the primary analyses above, to examine changes in the secondary measures from baseline to post-treatment and follow-up. Consistent with ***Hypothesis 3***, we also expect that benefits will be maintained over the two-month follow-up period.

### Exploratory analyses for potential mediators and moderators

We will also examine potential mediators and moderators of treatment outcome. Regression analyses will be conducted to determine whether sociodemographic or clinical characteristics mediate or moderate treatment outcome. We will follow the procedures outlined in Cohen and Cohen [75] to calculate residualized change scores for the SF-36, PDI and the HAQ. Multiple regression analysis will then be conducted with outcomes separately; change scores (baseline minus post-treatment) will be regressed onto a set of predictor variables including group (yoga vs. control), the mechanism of interest (e.g., self-efficacy) and the interaction between the mediator/moderator and group. A significant interaction term indicates moderation [76] and post-hoc analyses will be conducted to examine the moderator variables. If evidence of mediation is found using the traditional Baron and Kenny approach, the Sobel test, a statistical test for mediation will be conducted to yield a more precise picture of mediation. Exploratory analyses will be conducted in the data derived from the weekly monitoring forms; for example, we will examine whether changes in overall physical activity may moderate treatment outcome.

### Qualitative analyses

Digitally recorded qualitative data will be transcribed and entered into computer files. Data in these forms consist of large verbal texts that must be interpreted, organized, and summarized using a text-based analysis technique. The technique most appropriate to our data and research goals is ethnographic content analysis (referred to as template text analysis by Crabtree and Miller, 1992) because we wish to systematically integrate it with the close-ended HRQOL and functionality data. This technique involves the creation of a codebook based on variables identified by the researcher as relevant to the research questions and goals. Texts are read and codes are assigned to relevant portions of the text and entered. The process is iterative, however, and code categories are revised, expanded, and created as research progresses. This requires that coding proceeds from the very beginning of the research and updating of previously coded materials is carried out as the coding is refined.

We will employ Dedoose, one of the most effective qualitative data analysis tools currently available. Employing a web-based interface, Dedoose was designed primarily for team-based research projects and includes features that allow for efficient coding, integration with categorical and scale data, and database searching and retrieving. Unique to Dedoose is the ability for research team members with security clearances from the PI to access the data. All transfer of project data is encrypted for security. Further, the process of identifying and exploring coding patterns is automated via easily generated tables and user-defined output is available at any time in MS Word or Excel formats.

The process of coding allows for the thematic analysis of data. To address inter-rater reliability concerns, we will use multiple coders and measure intercoder agreement (the extent to which a group of individual coders concur in their identification of themes and codes). Thus, we will be able to ensure a high level of reliability in our qualitative data analysis.

### Integrating methodologies

For this study, the qualitative interviews derive a number of themes from the HAQ and the PDI. Our intention in doing so is to delve into how pain limits the HRQOL of young people with RA in much greater depth than the quantitative measures alone allow and to triangulate the findings. The qualitative instrument seeks to capture in detail the nuances of the impact of RA on the respondents' life experiences in seven domains: 1) family/home responsibilities, 2) recreation (hobbies, sports), 3) social activity, 4) occupation, 5) sexual behavior, 6) self-care, and 7) life-support activity (eating, sleeping, breathing). We will explore in detail the nature of the subjects' symptoms and what life domains the symptoms affect, how specifically these life domains are impacted, the patients' attempts at self-management of the symptoms, impact of the symptoms on these networks, and the patients' perceptions of the effects of the yoga program.

The integration of qualitative and quantitative methodologies represents a promising, though largely underutilized, avenue for researchers. An emergent and compelling body of literature supports the value of mixed methods [77-80] and suggests a panoply of means by which the techniques can be effectively combined. In this study, we are electing to administer quantitative measures as well as a qualitative measure that derives its basis from the quantitative measure in order to: a. cross-validate the qualitative and quantitative data (triangulation), b. clarify the data from one method with the data of another (complementarity), and c. broaden the depth of the data (expansion) [79]. Overall, we intend to optimize our understanding of the effects of the IYP for this population; we believe this integrated methodological approach will yield a much richer set of data than if we were to follow either strategy alone and distinguishes our proposed study as unique.

## Protection of subjects

This study was approved by the South General Institutional Review Board within the Office of the Human Research Protection Program at UCLA (IRB Number G08-02-045-03). The study will be carried out in compliance with the Helsinki Declaration.

### Potential risks

Yoga is generally regarded as safe and adverse effects are rare, especially within the Iyengar program in which props are used for support and teachers undergo vigorous training. A number of case reports have indicated adverse events following yoga, specifically, the presence of subcutaneous emphysema [81] and shortness of breath and chest pain [82]. However, these events were in response to intensive *pranayama*, or breathing exercises, with the shortness of breath case experiencing difficulty following *kapalabhati pranayama*, involving strong forced breaths which may push the body to physiologic extremes [82]. Such intensive breathing techniques will *not *be used in this yoga intervention. Back bending postures, such as those used in IY can be harmful if conducted without progressive musculoskeletal actions [83], and great care will be taken in developing the yoga program to ensure that muscles are not stressed. A qualified assistant in addition to the instructing teacher will also be present during all sessions to ensure that patients are completing the poses correctly. Participants may feel uncomfortable disclosing personal information about their mood and symptoms on study questionnaires and during interviews. The 28 joint count medical exam and blood draws may cause some temporary discomfort at the time of examination.

### Protection against risks

The major potential risks from the yoga class are musculoskeletal strains or injuries. We will minimize these risks by conducting the yoga intervention in the safest manner possible. A progressive series of poses has been developed for this trial; this gradual progression tailored to the participants is designed to avoid musculoskeletal injury. In addition, props will be used to support participants in more challenging poses. The general safety parameters of the yoga program are: 1) heart rate will not exceed moderate training range (115 BPM) - we will ask participants to check their pulse at the end of each yoga session during a timed one-minute period and to report their heart rate; 2) attention will be paid to spinal safety; 3) balance will be carefully monitored (e.g., chairs are used to assist balance as needed). Students will be given modifications of postures using props to accommodate for individual differences and limitations, and receive individual attention if postures are too challenging. An assistant will work with the experienced IY teachers during all classes and will observe for any difficulties and assist participants as needed.

To minimize any potential risks or discomfort associated with study assessments, all assessments will be conducted in a private room. Participants will be encouraged to raise questions or concerns at any time and be reminded that they may withdraw at any time without penalty. A trained phlebotomist will collect all blood samples and a rheumatologist will conduct the medical exams.

Confidentiality will be maintained by the use of unique identification numbers. All data and participant lists will be kept in locked file cabinets with access restricted to research staff. All data forms will have subject ID only. No data on individuals will be published. All hard copies of the data will be kept in locked file cabinets, even after the completion of the study. Data obtained from participants will be stored in a computer database, which will be password accessible only. Password-protected access will be given only to the principal investigator and co-investigators on the study. Electronic data will be collected using a secure survey software and questionnaire tool called SurveyMonkey with many system safeguards such as password protection, IP-masking, and data encryption (Secure Sockets Layer or SSL), etc. The hypertext transfer protocol secure (HTTPS) URL provides encryption and secure identification of the server, as indicated by a lock icon, to ensure confidentiality. SurveyMonkey is one of the most common secure online questionnaire services used to survey research participants and has physical and environmental controls in place to protect data.

### Yoga safety and adherence monitoring board

The Project Director will oversee the process of data collection and ensure that data from the medical evaluation, self-report questionnaires, and interviews are reviewed for completeness and accuracy. Reviewed data will be secured in locked file cabinets in the project offices. During each of the treatment waves, we will carefully monitor any potential threats to the safety and well-being of participants. A board will be created with the purpose of monitoring instructor adherence to the study manual and safety of patients. The Board will review the notes taken by assistants at each session for a randomly selected sample of 20% of patients, to be equally distributed across those who receive the IYP first and those who receive the control condition first. Using these notes, the Board will rate each instructor on the extent of adherence to the yoga manual. The Board will also review regular safety reports that will include information regarding recruitment, demographics of subjects, and any possible safety concerns including adverse events.

## Discussion

Young people with chronic, disabling RA symptoms represent a significantly underserved population. The overarching goal of this project is to develop and test a novel prevention and risk reduction approach to improving functioning and enhancing the quality of life for this population. The data from this project will inform a larger study to conduct a more formalized and advanced trial to further evaluate the utility of IY for improving pain and functionality among RA patients. Ultimately, by amassing a strong evidence base, our IY treatment protocol may eventually become integrated into the established prevention and treatment approach for RA as well as the management of other chronic and debilitating conditions.

A number of limitations may pose challenges to carrying out the proposed study. Given that RA is more typically diagnosed in older adults, recruiting participants who meet our age criteria may prove difficult. It is possible that attrition rates will be higher than predicted. A significantly greater drop-out rate would contradict our hypothesis about feasibility and this finding would force us to reconsider our design. Another potential study limitation is possible difficulties in scheduling the IYP sessions to avoid conflict with school or work and childcare issues. Classes will be scheduled on weekends and evenings at times when the majority of participants are available to address this potential difficulty. Participants will also have the opportunity to attend one make-up session if they miss any regular group classes. Another potential limitation will be the development of an arthritic flare during the IYP or control period. If a participant develops a flare, he or she will be instructed to communicate with his or her treating rheumatologist. The principal investigator will set up a meeting with the Data and Safety Monitoring Committee, which includes a rheumatologist. The Committee will develop a modified treatment plan so that the participant can remain in the IYP and continue with the program using modified poses.

We recognize that the control group does not account for the group or social aspects of the IYP. However, we will assess the extent of the social support using qualitative methods to determine the extent to which these aspects may impact outcomes. The decision to include a wait-list control group was based on the consideration that including a group-based control intervention would be premature at this early stage of the clinical trial process. We plan to explore the initial feasibility, safety and efficacy of the IYP before moving on to a large-scale clinical trial employing a group-based control condition that controls for group effects related to the IYP. It is also possible that the results of the study will reveal null findings and no significant differences will emerge between the control and intervention groups. If this is the case, analyses will be conducted to determine possible causes of negative findings. Perhaps the intervention will only be effective for a sub-group (such as young adults) or for a limited number of outcomes. These findings will, in turn, inform future investigations of yoga for RA. Even if null findings occur, post-hoc analyses will prove valuable in refining further work in this area.

Despite these limitations, our study is an innovative contribution to the field of yoga research. One of the more novel aspects of the study is that we will use a mixed methodology assessment. This approach will allow us to learn more from the participants in ways that will help ensure the success of future studies and may lead to the formulation of additional hypotheses. A key feature of this project is the building of this study within a theoretical model that will guide us in testing specific pathways for IY effects, including the impact of psychospiritual aspects of IY on functional and HRQOL outcomes. These analyses represent the first step in the eventual delineation of salient moderators and mediators of the effects of yoga in this population. Based on the present findings, the model can be more fully explicated and tested in multi-center studies with larger samples.

## Abbreviations

ANOVA: repeated measure analysis of variance; ASES: Arthritis Self-efficacy Scale; BSI-18: Brief Symptom Inventory 18; CPAQ: Chronic Pain Acceptance Questionnaire; CRP: C-reactive protein; CSQ: Coping Strategies Questionnaire; DAS: Disease Activity Scale; DMARDs: disease-modifying rheumatic drugs; ESR: erythrocyte sedimentation rate; FACIT-Fatigue: Functional Assessment of Chronic Illness Therapy Fatigue Subscale; FFMQ: Five Factor Mindfulness Questionnaire; HAQ: Health Assessment Questionnaire; HRQOL: health-related quality of life; HTTPS: hypertext transfer protocol secure; IL-1 RA: interleukin-1 receptor antagonist; IL-6: interleukin-6; IY: Iyengar Yoga; IYP: Iyengar Yoga Program; NRS: numeric rating scale; NSAIDs: non-steroidal anti-inflammatory drugs; PDI: Pain Disability Index; PSQI: Pittsburgh Sleep Quality Index; RA: Rheumatoid Arthritis; RCT: randomized control trial; SF-36: short form 36; SSL: Secure Sockets Layer.

## Competing interests

The authors declare that they have no competing interests.

## Authors' contributions

SE, JT, SS and LZ participated in the conception of the trial and in plans for the data analysis. SE, JT, SS, LZ, LC and BS drafted the manuscript. BS participated in the design of the study intervention. All authors read and approved the final manuscript.
